# Exhausting, but necessary: the lived experience of participants in an intensive inpatient trauma treatment program

**DOI:** 10.3389/fpsyg.2024.1341716

**Published:** 2024-05-28

**Authors:** Veronica Vaage-Kowalzik, Jeanette Engeset, Marianne Jakobsen, Wenche Andreassen, Julie Horgen Evensen

**Affiliations:** ^1^Division of Mental Health and Addiction, Oslo University Hospital, Oslo, Norway; ^2^Private Practice, Oslo, Norway

**Keywords:** PTSD, EMDR, PE, therapist rotation, intensive trauma treatment, qualitative research, patients' perspective

## Abstract

**Background:**

Intensive inpatient treatment programs have shown robust results in the treatment of post-traumatic stress disorder (PTSD). How patients experience this treatment program and what changes they experience as a result of the treatment have, however, only scarcely been explored through qualitative studies.

**Objective:**

This study aimed to explore the lived experience of participants in an intensive inpatient trauma treatment program. Our research questions were as follows: how do patients experience intensive trauma-focused treatment? How do they experience possible changes related to participating in the treatment program?

**Methods:**

Six patients diagnosed with PTSD with significant comorbidities, who recently participated in an intensive 2-week (4 + 4 days) inpatient trauma treatment program with prolonged exposure (PE), eye movement desensitization and reprocessing (EMDR), and therapist rotation (TR), were interviewed with a semi-structured qualitative interview. Transcripts were analyzed using a thematic analysis approach.

**Results:**

Our analysis resulted in five main themes: (1) the need to feel safe; (2) the benefits of many and different therapeutic encounters; (3) variable experience with elements of treatment; (4) intensity; and (5) experienced change. Our results suggest that feeling safe within the framework of the treatment program facilitated the treatment process. Many and different therapeutic encounters, both through TR and with ward staff, contributed to experienced change. All participants described the intensity as facilitative to trauma processing. However, most participants also describe often feeling too overwhelmed to benefit from all elements of the treatment program.

**Conclusions:**

Our findings suggest that participants experience the overall treatment program as beneficial and contributing to experienced change. Participants described the intensity of the program as exhausting, but necessary. Most did, however, report at times of being too overwhelmed to benefit from elements of the program. Consequently, our results prompt us to question the optimal level of intensity.

**Trial registration:**

ClinicalTrials.gov identifier: NCT05342480. Date of registration: 2022-04-22.

## 1 Background

“The cure for pain is in the pain.” This quote by the 13^th^-century Persian poet Rumi takes on particular significance when exploring patients' experiences of current-day trauma treatment. Trauma-focused therapy, particularly confronting traumatic memories, is known to be demanding and painful. Consequently, we need better knowledge of how trauma-focused treatment is experienced to know if enduring the pain justifies the possible gains. Post-traumatic stress disorder (PTSD) leads to significant subjective suffering and often limits vocational and social functioning. It is associated with increased risk of suicide, poor physical health, and significant psychiatric comorbidity (Bisson et al., 2015), thus burdening both the patient and society (Kessler et al., 2017; Watson, 2019). The World Health Organization's (WHO) guidelines for PTSD treatment (WHO, 2013) recommend cognitive–behavioral-based treatments for PTSD, such as prolonged exposure (PE) (Foa et al., 2021) and eye movement desensitization and reprocessing (EMDR) (Shapiro, 2018). A recent systematic review of treatments for PTSD confirms these recommendations (Lewis et al., 2020a). Previous quantitative research studies have compared trauma-focused to non-trauma-focused treatment, finding the strongest effect for PTSD in trauma-focused treatments (TFT) (Bradley et al., 2005; Bisson et al., 2013; Cusack et al., 2016). However, dropout rates are significantly higher for trauma-focused therapy than for non-trauma-focused therapy (Lewis et al., 2020a). Lewis et al. suggest that this disparity is related to the patient's ability to tolerate their focus on traumatic events (Lewis et al., 2020b).

During the last 10 years, intensive trauma-focused treatment programs (ITTPs) have been developed. A systematic review of intensive empirically supported treatments for PTSD containing treatment modalities including PE and EMDR found that these intensive treatments have a higher rate of treatment completion and suggests that intensive treatment for PTSD may be an effective alternative to standard treatment to prevent dropout (Sciarrino et al., 2020). The current study models itself on the inpatient ITTP developed at the Psychotrauma Expertise Center (PSYTREC), Netherlands (Van Woudenberg et al., 2018). This program includes daily EMDR and PE sessions, physical activity (PA), psychoeducation groups (PEG), and therapist rotation (TR) (Van Minnen et al., 2018). This research group has published several studies documenting robust treatment results for patients with PTSD (Van Woudenberg et al., 2018), including those with comorbid disorders (Kolthof et al., 2022; Paridaen et al., 2023), and for those with the dissociative subtype of PTSD (Zoet et al., 2018). In a recent Swedish feasibility study, a similar intensive treatment program using PE and EMDR reported significant reductions in PTSD symptoms (Gahnfelt et al., 2023). However, none of these research groups nor other researchers have qualitatively explored how intensive multicomponent inpatient trauma treatment is experienced by participants. A public regional outpatient unit for trauma treatment in Trondheim, Norway, has implemented a version of the Dutch intensive treatment program and adapted it to their outpatient facility with good results (Auren et al., 2022). This research group reported, through a qualitative study, that patients found the treatment program very demanding but worthy in terms of symptom reduction. The intensity of the treatment and TR were experienced as important for treatment efficacy, and the sense of unity with other participants and PA were both factors that facilitated easier completion of the program (Thoresen et al., 2022). Furthermore, two qualitative studies have described intensive trauma-focused therapy programs with one treatment modality. In a written survey of veterans with PTSD receiving a 2-week massed PE treatment program, Sherrill et al. (2022)found that treatment engagement was sustained through quick and meaningful symptom relief and that treatment was prioritized and avoidance-limited. Kovacevic et al. (2023) reported that participants in a 1-week massed Cognitive Processing Treatment program described changed PTSD symptoms and improved cognitive and affective coping skills.

Non-intensive TFT has been the subject of inquiry in some qualitative research studies. A recent systematic review encompassing nine studies (seven PE or trauma-focused cognitive behavioral therapy) synthesized the findings into four primary domains representing the temporal sequence of TFT: overcoming ambivalence toward TFT, experience of treatment elements, motivation for dropout or retention, and perceived changes post-treatment. The authors summarize that, though patients report high levels of distress and re-emergence of symptoms during treatment, most perceived the hardship as essential for improvement (Gjerstad et al., 2024). The review incorporates only one study on EMDR; it does, however, reference an earlier systematic review that examines five EMDR studies (only one with PTSD as the main focus of treatment). The author emphasizes the patients' perception of safety as a necessary condition for EMDR to be effective and explores changes including coming to terms with the past, cognitive and behavioral changes, and core “transformational” changes experienced by patients as resulting from EMDR (Whitehouse, 2021). Both reviews highlight the significance of qualitative studies that incorporate the patient's perspective, which deepens our understanding and enhances the effectiveness of trauma-focused treatments.

In summary, there is robust evidence for the treatment effect of ITTP. This intervention has, however, scarcely been investigated through qualitative analysis, and little is known about how patients experience intensive inpatient multicomponent trauma treatment. The current study aimed to explore patients' lived experience of a 2-week intensive inpatient trauma treatment program combining EMDR, PE, PA, PEG, and TR. Our research questions were as follows: How do patients experience intensive trauma-focused treatment? How do they experience possible changes related to participating in the treatment program?

## 2 Methods

### 2.1 Design, ethics, and data collection

The present study was conducted in a public psychiatric-combined inpatient and outpatient clinic in Oslo, Norway. The clinic offers treatment for a wide range of mental illnesses and is part of the specialist healthcare system that requires patients to be referred by a doctor. All patients were recruited from this clinic. The current study is part of the ongoing Norwegian intensive inpatient trauma treatment pilot project. Intensive combined treatment with EMDR and PE has not been previously conducted in an inpatient setting in Norway. The main aim of this pilot project is to examine if ITTP is feasible in a regular public healthcare setting. A total of 18 patients participated in the parent feasibility study, in three groups of six patients, receiving treatment between 2021 and 2023. The current study describes the experiences of the second group of patients being treated. The results from the feasibility study have not yet been published as follow-up is still ongoing.

The Central Norway Regional Ethics Health Committee (REC South East 0704/2022) has approved the study, which also includes the qualitative interviews. Clinical Trial gov. identifier 453358. Written informed consent was obtained from all participants.

### 2.2 Participants and procedure

All patients (*N* = 6) from the second treatment group in the pilot study were invited and consented to participate in the nested qualitative interview. All six patients had completed the program and participated in all therapy sessions. The pilot study treatment program had the following inclusion criteria: patients had to fulfill diagnostic criteria for PTSD diagnosis, have had >1 traumatic life event, have had symptoms lasting >6 months, and have had at least one prior psychotherapeutic treatment (>3-month duration). Patients had to be in the age range of 18–65 years and should speak a Scandinavian language adequately. The exclusion criteria were patients having a psychotic or bipolar disorder, those with active substance abuse issues, individuals currently in a abusive or life-threatening situation, or those attempted to take their own life in the past 3 months before treatment. All participants were included from our outpatient clinic. Before referral, patients had been diagnostically assessed with a structured clinical interview, the Mini-International Neuropsychiatric Interview (MINI) (Sheehan et al., 1998), and even with the Structured Clinical Interview for DSM-III-R (SCID-II) (Williams et al., 1992). After referral, patients were invited to an assessment interview at the inpatient treatment facility where they were given further information about the treatment before giving informed written consent. After inclusion, patients were prepared for treatment through a 4–5-day pretreatment admission where a detailed, individual treatment plan was devised (trauma timeline with index traumas and *in vivo* exposure plan), and patients received psychoeducation based on the treatment rationale.

Participants in the current study were six women aged 21–45 (average 28, median 25) years. All of them had previously received therapy for 1 year or more, among which five had engaged the public psychiatric health service since their mid-teens or early 20s. Four had been in contact with the child protection services and/or child psychiatric services due to psychological or physical abuse, neglect, and/or parents with substance abuse issues. All of them reported multiple traumas. All but one had experienced sexual assault. All had experienced systematic bullying and/or recurrent humiliating verbal assault. All patients had current comorbid psychiatric diagnoses including attention deficit disorder (Bisson et al., 2015), personality disorders (Watson, 2019), other dissociative (conversion) disorders (Kessler et al., 2017), and autism spectrum disorder (Bisson et al., 2015).

In an explorative feasibility study of this kind, a smaller participant pool is considered sufficient for thematic analysis (Braun and Clarke, 2006; Clarke et al., 2015). Interviewing six patients was thus deemed acceptable by the authors.

### 2.3 Treatment

**Figure 1:**
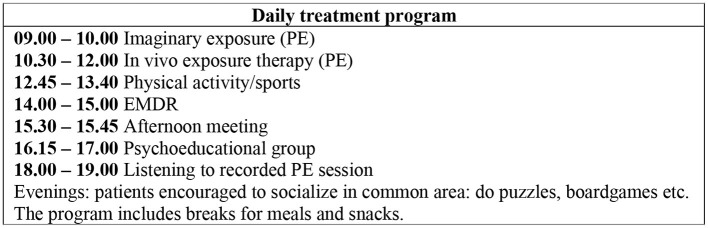
Daily treatment program.

The treatment program had a 2-week time frame with 8 full-treatment days (Monday–Thursday) and 2 days (Fridays) with team meetings and planning. Patients went home during the mid-treatment weekend. The program contained daily PE and EMDR sessions, between-session PA, and PEG (see Figure 1). A multidisciplinary team was provided to each patient. TR was used for EMDR and imaginary exposure (part of PE). The patients thus met 11–12 different therapists during the treatment. The program models itself on the PSYTREC program in the Netherlands (Van Woudenberg et al., 2018) but contains fewer PA and shorter EMDR sessions. Rather than focusing on PA, we have, similar to the Trondheim trauma treatment program (Brynhildsvoll Auren et al., 2021), expanded elements of PE by both listening to imaginary exposure (IE) session recordings and using *in vivo* exposure (IVE). IVE as an element of PE was implemented as it targets avoidance behavior particularly relevant for this patient group (Cusack et al., 2016).

Both IE and EMDR were carried out by psychologists and psychiatrists trained in the treatment methods. IVE, PA, PEG, and, when needed, assistance while listening to the recorded PE sessions were carried out by ward staff. Ward staff were also available for supportive *ad hoc* counseling. The ward staff consisted of nurses (some specialized in psychiatric nursing) and social workers. All permanent staff had received trauma treatment courses, including a course in PE. Therapists and representatives from ward staff had a daily 1-h meeting to ensure adherence to the treatment protocols (Shapiro, 2018; Foa et al., 2021) and to monitor progress and plan the following sessions. On-site supervision was provided in PE and EMDR therapy by qualified supervisors to further ensure treatment adherence. All patients remained under the primary care of their outpatient therapists prior to and after their treatment admission.

### 2.4 Data collection

A semi-structured interview was designed to explore patients' lived experience of the treatment. A semi-structured interview ensures thematic equality between interviews while still allowing a flexible exploration of the main themes (Kvale and Brinkmann, 2009; Qu and Dumay, 2011; Alvesson et al., 2020). The interview guide was developed by the pilot study research group. It consisted of seven main questions, with follow-up questions to deepen and broaden the main questions (see Table 1). Focusing on the experience of the treatment program, the patients were asked about both helpful and unhelpful elements of the treatment and further asked specifically about various treatment elements. Patients were asked about changes in cognitions, feelings, and bodily awareness as well as changed interpersonal relationships and changes in everyday behavior with respect to possible experienced change during or after treatment. The interview lasted 30–45 min and was conducted at the inpatient treatment facility. All patients were interviewed 1–2 weeks after finishing the treatment program by interviewers independent of the treatment program and research project. Three interviewers shared the task between them, and two were present at each interview. Before the interviews were conducted, the patients were told that the interview was going to be centered around their experiences, both good and bad, of receiving the treatment to learn from the patients and thus be able to offer better care. The patients were informed that the interviews would be transcribed and anonymized. The interviewers made an effort to create a supportive, informal tone, encouraging the participants to elaborate freely on relevant themes. The interviewer followed up with in-depth or clarifying questions when needed. The interviews were audio-recorded. A research assistant transcribed the interviews and anonymized all transcriptions. Due to a fault with the recording equipment, one interview was not recorded. This interview is, therefore, not included in the data material.

**Table 1:** Examples of questions asked in the interview with patients.

**Topics**	**Questions**
Today's situation and need for treatment	How have you been since completing the treatment program? Has it been necessary for you to receive any treatment or additional follow-up since discharge?
Preparations for the treatment	Did you feel that you were adequately prepared for the treatment? Was there anything that could be done to make you better prepared?
During treatment	How did you experience the treatment you received in the inpatient ward? Do you think you changed in any way during, or after, the treatment? If so, how (changed thought patterns, emotions, bodily experiences, new insights, or altered relations)? Do you remember a special situation, event, or episode that was particularly important to you?

### 2.5 Analysis

We used thematic analysis, as described by Braun and Clarke (2006), to explore and analyze the material. This method identifies recurrent themes and patterns of meaning-making, is suited to analyze the experiences and meaning-making of participants, and is thus suitable for the current study. We identified themes or patterns within the data by using an inductive bottom-up approach—not trying to fit the data into a pre-existing frame (Patton, 1990). To better explore the lived experience and meaning-making of the participants in the program, we used the hermeneutical–phenomenological position (Gadamer, 2013). We conducted the analyses in six phases described below to increase the traceability (Castleberry and Nolen, 2018). Phase 1: The first author compared audio recordings of the interviews to transcriptions made by a research assistant to ensure the quality of the transcription. Phase 2: All authors read all transcripts looking for answers to the research questions. We explicitly looked for both negative and positive experiences. The first and second author generated initial codes and searched for themes independently and later compared and discussed their findings with the last author. To ensure correspondence between raw data and codes, specific words and syntax from the transcriptions were used when identifying and labeling various aspects of the data material. Phase 3: Codes were reorganized into possible themes. The last author gave feedback on the first and second author's reduction and initial thematic categorization, further refining the themes and subthemes through discussion. These three authors discussed their unique understanding of the material and critiqued the categorization conducted so far. Phase 4: The first, second, and last author reorganized and refined the themes and subthemes, comparing it to the initial coded data to ensure that the analyses were consistent with the information from the interviews. Codes under different themes were compared to each other to ensure right fit, and re-categorization was carried out where necessary. Phase 5: All authors reviewed themes and subthemes presented listwise, discussed their relevance for the research questions and the research project, and then agreed on the current categorization and presentation. The described process made our interpretations less dependent on individual preferences (Malterud, 2011). Phase 6: Each theme was described in a report that emphasized the core or essence of the theme. Participants' quotes were added to affirm discussion and arguments. Documents were used to track the process of coding and generation of themes.

The authors have different therapeutic orientations. VV-K is both a PE and an EMDR therapist; MJ, JHE, and WA are EMDR therapists; MJ is also a certified EMDR supervisor; and JE has no specific therapeutic orientation. This diversity has hopefully broadened and enriched our interpretation of the data. This transparency is made in accordance with the checklist of reporting qualitative research by Tong, Sainsbury, and Craig (Tong et al., 2007). We used the labels general, typical, and variant to indicate the recurrence and representativeness of therapists' experiences, as suggested by Hill et al. (2005). When something was mentioned by all, it was labeled as general and in the text referred to as “all patients.” When something was mentioned by more than half the patients, it was considered typical and in the text referred to as “most patients.” We use the expression “some patients” when something was found to be a variant represented by less than half the patients but more than one.

## 3 Results

We organized the material into five main themes: (1) the need to feel safe, (2) the benefits of many and different therapeutic encounters, (3) variable experience with different elements of treatment, (4) intensity, and (5) experienced change. The themes and subthemes, including representative quotes from patients, are summarized in Table 2. All names are pseudonymized.

**Table 2:** The identified themes and subthemes with quotes from the patients.

**Themes**	**Subthemes**	**Examples of quotes**
The need to feel safe	Getting prepared for treatment Access to ward staff	“The pre-treatment admission taught me a lot” Merete “There was always someone there if you needed someone” Louise
The benefit of many and different therapeutic encounters	Added perspectives and focus on own process through therapist rotation New relational experiences	“It was really good to rotate the therapists. To get several perspectives.” Kari “He was really good at setting me straight, almost like a parent (laughs).” Kristin
Variable experience with elements of treatment	What worked for one was difficult for another The total package was good	“To me, that (the listening) was what made me remember stuff.” Kristin vs. “It (the listening) was awful (…) I got so self-critical.” Louise “I just think the total package was great.” Sigrid
Intensity	Exhausting, but necessary Too intensive? Being back on my own	“Because when I have been in therapy previously, I haven't managed to… to connect to my reactions. But like here, like, here you, in a way, had no choice.” Merete “I was so exhausted that when I sat there. It (the psychoeducation) went in the one ear and out the other.” Kristin “So the difficult thing is to find the balance between the tools I have learned for 2 weeks and the life I have lived prior to that.” Kari
Experienced change	Acceptance and ownership to one's trauma history Less numbness and dissociation, greater access to feelings Changed personal relationships Self-compassion and grief	“And the fact that when we talked through the trauma, I had very few pieces of the puzzle, but they helped me fill in the rest of the picture.” Merete “I feel that the fear is stronger because I don't dissociate from it. And that's really a good thing, but it is, like, really tiring.” Kristin “I have had to break off with a lot of people, and now it's really empty.” Kristin “I think it has become much less guilt and more grief.” Sigrid

### 3.1 The need to feel safe

All participants emphasized the importance of feeling safe on the ward and with ward staff and/or with therapists. Feeling safe allowed them to enter the treatment process and stick with the treatment program. Being adequately prepared for the program emerged as an important aspect of feeling safe.

#### 3.1.1 Getting prepared for treatment

All participants emphasized the importance of getting adequately prepared for treatment and that the pretreatment admission was important for this purpose. Most participants described that getting to know the ward staff and the multidisciplinary team made them feel safer. Kristin said: “To me, the week (pre-treatment admission) was very important…I felt a need to know the place… and the people. Because I have always felt unsafe around other people. So this (the ward) was really the first place where I, like, have felt safe around other people. Adults. Therapists and so on…” Furthermore, learning about the treatment program and the rationale for treatment was emphasized as important. Merete said: “...But I absolutely felt that I got more out of the treatment because I knew, I knew for example exactly how I was going to talk about the memories. Because we had sort of practiced it in the pre-treatment admission.” Most patients, however, said it was impossible to be prepared for how tough it was going to be.

#### 3.1.2 Access to ward staff and multidisciplinary team

All participants emphasized how the ward staff made the treatment program feel safe. Most said that it was good to know that staff were available if they were struggling and that the ward staff knew what they were going through. Kari said: “…And when I experienced a lot of emotions, it didn't really matter who was my primary contact (ward staff), I just made eye contact with one of them and, like, ‘*Now we need to go and talk*…' And it is really good that everyone knows stuff. That they write reports, etc.” Having a multidisciplinary team of two staff members and a therapist was pointed out as important by most participants. Patients described the team as an anchor, a family, or as a safety net. Merete said: “So they (two staff members from the team) were really good to have, and they also helped me when I was not doing well. But, it sometimes happened that they were not working, and then I got (assigned) a new member of staff that I maybe had never met before, and did not know anything about, and then I didn't feel totally comfortable asking for help, if I, for example, was dissociating (…) But, I thought that all (staff) were competent, and it was good that there was always someone to you could approach.”

### 3.2 The benefit of many and different therapeutic encounters

All participants described how the treatment program had facilitated both new insights and meaningful encounters with ward staff and therapists. Most emphasized how this positively differed from previous psychotherapeutic treatments.

#### 3.2.1 Added perspectives and focus on own process through therapist rotation

All but one expressed enthusiasm with regard to TR. Most described how TR gave them added perspectives. Some patients experienced that TR made it easier to focus on their own process rather than on the relationship to the therapist and that not being so close made it easier to open up and talk about difficult experiences. Merete said: “I thought it turned out really good, that we changed (therapist) often and that it… like I said… I came to feel like it was my process in a way. Rather than it being me and another that were going to go through it together. It also made it feel a little less - because it is really very private and personal stuff that one talks about – I think it would have felt a bit, like, creepy if, like, it was just one person I was to talk to about all those most difficult things…” Kari also described how TR made the therapeutic process less dependent on the quality of the relation to one's therapist: “I experienced it as really beneficial to have different therapists. I felt that, most probably, if I had ended up with only one therapist then we would have built a kind of relationship. And if the chemistry hadn't been right, then I think I would have had a really lousy experience. Because I have had very many different therapists (previously), and I have had a bad experience… So it was really good.”

The only patient who was negative to TR (Louise) said: “To me, it was difficult that we, like, changed therapists every therapy session. To me, it made the treatment a bit harder, because I had to relate to a new person every time (…) So that, I was a bit like, I should have liked it to keep to one or two, maybe three therapists that rotated.”

#### 3.2.2 New relational experiences

All the participants described new relational experiences. Most struggled to trust people in general, particularly men. During the treatment program, most participants experienced that more people than they had previously thought were trustworthy. Kristin emphasized how TR gave her new relational experiences: “I think it's really smart (therapist rotation), because I have very little trust (in people), because of specific things, so that helped me see that there are several people, like, that are good. And that people are, kind of, safe. So it was good to have several (therapists).”

Some described that a staff member that they struggled to trust in the beginning (because of his gender or because he/she reminded her of someone from the past) after some days became a safe person. Some described staff members as role models they had lacked in their upbringing. Kristin said: “And also it was just generally good to understand that it exists, just to see what adults really should be like. Because I have not seen it or experienced it. So I got a really, like, (new) perspective on my childhood and my parents (…) But one has to experience it to really understand it, cause one can hear it as much as that, but one has to experience it.” These experiences often lead to an understanding of what they had missed out on in their upbringing.

For some, it was a new experience to open up and expose vulnerability. Kari said: “…but also the first time I got a real big panic attack with someone present, (previously) I have always been by myself. And that was real, like, exposure, to show someone (the panic attack). It sounds like such a cliche, but to, like, show someone your worst side.”

### 3.3 Variable experience with different elements of treatments

All patients found two or more useful elements in the daily treatment program, but the participants disagreed about what was beneficial. What was helpful to one was challenging or even experienced as impeding to another. All patients, however, agreed that the treatment program overall was good.

#### 3.3.1 What worked for one was difficult for another

All participants described EMDR as beneficial, and all described gains from elements PE. Most participants described that imaginary exposure (IE) helped them either retrieve details of trauma memories or gain access to emotions. Louise, on the other hand, was struggling with IE. She, however, found *in vivo* exposure helpful: “I experienced really good progress when we were going outside to do exposure (…) There I managed to keep up, and make it to the next level…”

Most of the patients described the daily program as challenging, but well-structured. Kari said: “I enjoyed that we started with PE that I could listen to in the evening, and I liked that this was followed by *in vivo* exposure. And then EMDR in the afternoon. It was, kind of, a good set up.” Some wanted to swap around the elements of treatment in the timetable. Louise said she would have preferred to have IE later in the day and possibly EMDR as the first therapy session. Kristin would also have preferred to do EMDR as the first session, as this treatment often made her feel calmer, while she experienced IE as more anxiety-inducing and therefore thought she would have benefitted more from *in vivo* exposure if she did this part of PE after EMDR.

The patient group was most divided with regard to listening to PE recordings. Most of the patients described listening to the PE session as helpful, in that it was a different kind of trauma exposure and made them remember details of their trauma history or increase self-compassion. Sigrid said: “Yes, it was the especially the recordings. To listen to them, that really helped me. It was so surprising to me, that like, to just listen to yourself telling an awful story, that it helps one have self-compassion.” Kristin described how listening to recordings aided her memory “Ehm… I think it (the listening) went pretty well. It's just that one is pretty tired, and … but to me, that (the listening) was what made me remember stuff.” Some patients, however, struggled to listen to their recorded PE sessions as the listening made them self-critical and/or dissociative.

Most patients enjoyed the physical activity groups. Kari said: “The activity groups were good. I enjoyed... It was such a good variation. And the fact that we went for walks and got to know each other. Boxing and climbing were the definitive favorites. And Kubb in the garden, great fun. And you get to feel more secure about the ward staff too.” However, the group varied in the level of fitness, and some found it challenging to exercise in groups as it made them feel self-conscious.

#### 3.3.2 The total package was good

All participants described the overall treatment program as good or very good. Some of the participants described how different elements of treatment complimented each other and created a synergistic effect. Merete said: “And I think it was nice that, it was tiring, but it was nice to be able to get two so different types of therapy in the same day. Cause it kind of helped me, to first get a lot of stuff to the surface, and then later I felt that EMDR kind of helped me to contextualize and talk around it.” Kristin described: “I think talking about the trauma in the present tense (element of PE) helped me to remember (…) when I remembered more about how it was, and how I felt, the EMDR worked better too.” Kristin also described how listening to the PE recordings aided her memory and further benefitted her progress in the EMDR sessions.

### 3.4 Intensity

#### 3.4.1 Exhausting, but necessary

All the participants experienced the intensity of the treatment program as an important factor in the treatment process. Most experienced the intensity as necessary to stick to the processing of their trauma history. Louise said: “I think it was positive to kind of be very connected all the time. That there were no, like, long breaks between each treatment session. That it was easier in a way to reenter the trauma, or look back at things, because you were kind of in it—the milieu or the mindset.” Most described how the intensity of the treatment program made it difficult to close up. Sigrid explained how this allowed her to move on: “With regards to how many sessions we have had over those 2 weeks. It's almost like 6 months of normal treatment. How much further one has come, because one is vulnerable all the time. Because you can't, kind of, close up. And to put it in that perspective, it's like…Yes, but Hello! It's not strange that I am tired (…) because that was two bloody tough weeks, but it's so worth it.”

Some explained that the intensity helped them continue exposing themselves rather than avoid trauma triggers and related feelings. Merete described: “I think it's related to the fact that I didn't just have one therapy session and then was allowed to leave, and leave it behind, and avoid thinking about it.” She further described how the tight session schedule made her have a very intense psychological reaction early in the first treatment week and said “That made it very clear how inflamed it was (the trauma history), and what stuff I had to work through. So that became a tough…but also a really important experience.”

#### 3.4.2 Too intensive?

As described in the previous section, all patients described that the intensity of the program was beneficial and necessary to the therapeutic process. However, most patients also reported times where they were too overwhelmed to benefit from elements of the treatment program. This was particularly related to either listening to PE recordings and/or in psycho-educational groups. Some patients specifically reported that migraine and/or dissociative symptoms impeded listening to PE recordings. Some also reported a more general experience of being pushed over their preferred limit in or in between the therapy sessions. Kari said: “I had a really high SUD (Subjective Unit of Distress) and felt my body really resisting it. And to still be pushed (to do treatment), when I was completely destroyed. But I understand that is a part of the treatment, but I think, to me, that was the worst.” Only one patient however explicitly suggested changing the program and said she would have preferred to have 1 day free per week from therapy sessions.

#### 3.4.3 Being back on my own

Most of the participants described the transition from the 2 intensive weeks of treatment to returning home after discharge as tough and that they needed time to settle before going back to their normal chores. Most wished they had been better prepared for discharge to experience a smoother transition. Kristin said: “So I think, that was what was challenging when I got out of here, that suddenly you're back to everyday life, but your body is still in that bubble, in the treatment program. So I kind of needed a few days to just calm my nervous system, before I really had to, should have had to, go out to do stuff.” Most had started to regard their feelings and/or trauma history in a new way, and for some, that changed existing relationships, making the process of returning to these relationships difficult.

### 3.5 Experienced change

All participants expressed a change in how they related to their trauma history. All expressed being more emotionally connected and in touch with how they were feeling, most in a more nuanced and novel way than before commencing the treatment program. Most expressed that this influenced their day-to-day functioning and/or relationships.

#### 3.5.1 Acceptance and ownership to one's trauma history

All participants expressed that the treatment program had given them new insights related to their trauma history. Merete said: “... And that is something that, like, totally changed the understanding of my own experiences. And I feel that change in a very positive way. And that helps differentiate… differentiate between what actually happened, and in a way understand… understand what happened back then (…) Initially it was just about understanding what perspectives (of trauma) I had had. And then it was all about changing those perspectives. And that was, and I wasn't prepared for this at all, but that was very, very helpful, and I really think it has changed a lot.” Most participants explained how these new insights led to increased acceptance and ownership of their trauma history. Kristin said: “… what I remembered felt like it had been a dream. So, kind of, I had no ownership of what had happened. And that makes one really frustrated and confused and annoyed all the time. So, it helped a lot. And now knowing, when I am home, and I have a reaction, it's because of that memory, or that thing, like.” A greater sense of acceptance and ownership made it easier to separate what they should and should not take responsibility for. Sigrid said: “I feel like the most important thing regarding trauma is the sense of control. And if you take on all the responsibility, you feel a lot of control. Right? Because then …everything is on me. But it's been surprisingly freeing to think that ≪this happened, and there were loads of other, earlier things that happened, and it wasn't my responsibility, and someone else should have taken on that responsibility.”

#### 3.5.2 Less numbness and dissociation and greater access to feelings

All patients describe a greater ability to access a broader spectrum of emotions and a better ability to understand their emotional and physical reactions. Louise said: “I have, among other things, allowed myself to feel irritable and angry with certain people, something I never did before. That was also something we worked on to accept that it is ok (to feel angry).”

Most reported that less numbness and dissociative reactions made them gain access to a larger range of emotions. Merete said: “It made a big difference—that I was able to talk about and think about things that I previously could not think about without dissociating. (…) and it was still uncomfortable, but it wasn't at all as uncomfortable as it had been. And I notice that, in my body too, that there is less, like, the anxiety level has lessened...” Kristin explained how dissociation and numbness previously had worked as self-protective mechanisms: “I just think, like I said, just about my dissociation, it is…It's like a kind of shield, that I in a way left behind here.” Kari said: “Earlier I have always been shutting out everything. So now that I think a lot more, my brain has gotten a kind of kick-start. I feel that it is processing and works hard, like, compared to before when I couldn't even think, and was just laying around sleeping all day. So now I feel like it (the brain) has gotten some new energy, and then it's back working.” Both greater acceptance of their own trauma history and having greater emotional accessibility made some participants clearer about their needs and more likely to take these needs into account in their everyday setting. Merete said: “I value more…what I feel is right, like. And I value my own feelings and needs a bit more…”

#### 3.5.3 Changed interpersonal relationships

Most participants mentioned that new perspectives and insights related to their upbringing and trauma history affected their relationships with family, friends, or partners. Kristin said: “I can't view things the same way anymore. Like… I can't accept certain things from my family. Or, I want to be around my family, but it costs me a lot, like when people are screaming and those kind of things…So... That's been better, I have had to accept that I don't have a family I can lean on, and that I must lean on other things instead. To not have, like, not go around hoping… Cause I feel that that is what one does as a kid—hoping that things will get better, or that one can help someone, or…right. But I have tried breaking with that hope, but that has also been scary.” Some participants described feeling lonelier and that their lack of a proper non-professional support system had become more apparent.

#### 3.5.4 Self-compassion and grief

Most participants described how processing their trauma history had given them a more complete narrative and that by addressing feelings of guilt, shame, and responsibility for the experienced trauma, they had gained access to a more nuanced repertoire of emotions. This repertoire included feelings of grief as they more clearly saw themselves as victims of past traumatic events. Most thereby gained a new perspective on their upbringing and/or past relations.

Sigrid described her process like this: “It was during an EMDR session, I can't remember which. But… I told her about stuff. And… we went through it, and then she said ‘*Oh, that is so sad, and so painful that it happened.'* And that really hit me, because I never thought like that myself (crying). And I have had a very difficult childhood, and if only my mom had said that once, it would have helped me a lot. So it was a bit, like, odd, to think about. That just to hear it once is so intense. But it really helped me be kinder to myself. And like, it‘s painful and it's sad that it happened. But I can't change it, I must move on, and I can't pretend like it didn't happen…Because I had never heard such a thing. And to hear it several times, and in different ways, that has been really valuable.”

## 4 Discussion

This study aimed to explore the lived experience of participants in an intensive inpatient trauma treatment program. Our findings suggest that feeling safe within the framework of the treatment program, many and different therapeutic encounters, and the intensity of the program may contribute to experienced change. In the following sections, we discuss our findings in the light of therapeutic alliance, the level of intensity that is optimal for treatment benefits, and processes contributing to experienced change.

### 4.1 Feeling safe and establishing therapeutic alliance within the framework of the treatment program

To our knowledge, no previous qualitative study on inpatient intensive trauma treatment exists to date. Our patient sample described how accessibility to ward staff, a supportive multidisciplinary team, and pretreatment admission made them feel safe, something they emphasized as facilitative to the treatment process. All participants in our sample had significant comorbidities, and an inpatient facility likely provided a therapeutic framework that they experienced as perhaps necessary, or at least beneficial, to the therapeutic process. Therapeutic alliance is an established predictor of outcomes in psychotherapy (Flückiger et al., 2020; Howard et al., 2022). Recent quantitative and qualitative studies have reported that patients experience treatment alliance within massed trauma-focused treatment programs without TR (Goldfried, 1980; Galovski et al., 2022; Kovacevic et al., 2023). Our patient sample met new therapists nearly every day of the program and thus did not develop a one-to-one therapeutic alliance (bond) with the therapists. However, the patients developed an alliance (bond) with the multidisciplinary team and ward staff and a working alliance with the rotation therapists and treatment program itself.

One could imagine that this working alliance could enhance autonomy, as was explicitly mentioned by one patient who said TR made her feel that the process of therapy became *her* process rather than a shared experience with a therapist. One can hypothesize that this more autonomous process has a longer-lasting effect, continuing to be effective after completing treatment. Our findings support those of Van Minnen et al. (2018) who, in a quantitative study on patients in a similar intensive trauma treatment program, found that even patients with attachment problems could develop a good working alliance in a TR setting.

A recent narrative literature review that includes nine qualitative studies from diverse patient populations about their experiences of EMDR emphasizes the role of the therapeutic relationship (Marich et al., 2020). The authors discuss a study that compares the experiences of EMDR vs. eclectic therapy of victims of sexual abuse. This study reports that, while all the patients in the eclectic therapy group attributed their improvement to the therapeutic relationship, the EMDR patient group, though they spoke highly of their therapists, did not attribute their success in therapy to the therapeutic relationship but rather to the technical EMDR process and/or to how well the therapist followed the procedural protocols (Edmond et al., 2004). This finding is in line with a recent analysis by Hase and Brisch (2022) who used attachment theory as a framework for understanding how procedures and protocols in EMDR therapy are related to the therapeutic relationship. It could thus be that the therapeutic alliance with the therapists described by our patient group was partly a result of the EMDR treatment method.

Van Minnen et al. (2018) further found that 85% of patients preferred working with a TR team to being treated by one therapist only. Similar to this and the study by Thoresen et al. (2022), we found that patients were positive to TR and that TR facilitated new relational experiences and contributed to different perspectives. Expanding on these findings, we report that some patients experienced that changing therapists from session to session made it easier to focus on their own process rather than the relationship with the therapist and that not being so close made it easier to open up and talk about difficult past experiences.

Similar to Thoresen et al., we found that TR can provide corrective emotional experiences. These experiences are known to have curative potential in psychotherapy (Goldfried, 1980). Our sample reported experiencing being seen and understood by therapists and being able to trust and feel safe, experiences which they said were missing in their upbringing. Most patients reported similar corrective emotional experiences from ward staff, an aspect that is lacking in outpatient treatment.

To our knowledge, no previous qualitative study on inpatient intensive trauma treatment exists to date. Our patient sample described how accessibility to ward staff, a supportive multidisciplinary team, and pretreatment admission made them feel safe, something they emphasized as facilitative to the treatment process. All participants in our sample had significant comorbidities, and it is likely that an inpatient facility provided a therapeutic framework that they experienced as perhaps necessary, or at least beneficial, to the therapeutic process. Therapeutic alliance is an established predictor of outcomes in psychotherapy (Flückiger et al., 2020; Howard et al., 2022). Recent quantitative and qualitative studies have reported that patients experience a treatment alliance within massed trauma-focused treatment programs without TR (Goldfried, 1980; Galovski et al., 2022; Kovacevic et al., 2023). Our patient sample met new therapists nearly every day of the program and thus did not develop a one-to-one therapeutic alliance (bond) with the therapists. It seems like they, however, experienced an alliance (bond) with the multidisciplinary team and ward staff and a working alliance with the rotation therapists and treatment program itself.

One could imagine that this working alliance could enhance autonomy, as was explicitly mentioned by one patient who said TR made her feel that the process of therapy became *her* process, rather than a shared experience with a therapist. One can hypothesize that this more autonomous process has a longer lasting effect, continuing to be effective after the completion of treatment. Our findings support those of Van Minnen et al. (2018), who, in a quantitative study on patients in a similar intensive trauma treatment program, found that even patients with attachment problems could develop a good working alliance in a TR setting.

Van Minnen et al. (2018) also found that 85% of patients preferred working with a TR team to being treated by one therapist only. Similar to this study and the study by Thoresen et al. (2022), we found that patients were positive to TR and that TR facilitated new relational experiences and contributed to different perspectives. Expanding on these findings, we report that some patients experienced that changing therapists from session to session made it easier to focus on their own process rather than on the relationship with the therapist and that not being so close made it easier to open up and talk about past difficult experiences.

A narrative literature review including nine qualitative studies from diverse patient populations about their experiences of EMDR therapy emphasizes the role of the therapeutic relationship (Marich et al., 2020). The authors include and discuss a study that compares the experiences of EMDR vs. those of eclectic therapy undergone by victims of sexual abuse. They report that, while all the patients in the eclectic therapy group attributed their improvement to the therapeutic relationship, the EMDR group patients, though they spoke highly of their therapists, did not attribute their success in therapy to the therapeutic relationship but rather to the technical EMDR process and/or to how well the respective therapist followed the procedural protocols (Edmond et al., 2004). This observation is in line with that of a recent analysis by Hase and Brisch (2022) who used attachment theory as a framework for understanding how procedures and protocols in EMDR therapy are related to the therapeutic relationship. It could thus be that the therapeutic alliance expressed by our patient group was, in part, a result of the EMDR treatment method.

Similar to the study by Thoresen et al., we found that TR can provide corrective emotional experiences. These experiences are known to have curative potential in psychotherapy (Goldfried, 1980). Our sample reported experiences of being seen and understood by therapists and also being able to trust and feel safe, experiences which they said lacked in their upbringing. Most patients reported similar corrective emotional experiences from ward staff, an aspect that is obviously lacking in out-patient treatment.

### 4.2 What is the optimal level of intensity?

Previous qualitative studies describe how intensive trauma treatment facilitates commitment to treatment and limits avoidance (Sherrill et al., 2022; Thoresen et al., 2022). Similarly, our sample described that the intensity of the treatment program facilitated processing their trauma history, as the program gave little opportunity to close up emotionally between sessions. However, most patients also reported times when they were too overwhelmed to benefit from elements of the treatment program. Some further reported a more general experience of being pushed over their preferred limit in or in-between the therapy sessions. Our results thus raise the question of what the optimal level of intensity is and if patients would profit equally well, or better, from a somewhat less busy schedule or a slightly longer admission with more breaks. An outpatient study where veterans were administered cognitive processing therapy compared a 3-week to a 2-week program. In the 2-week program psychoeducation, mindfulness and yoga were omitted, but the number of therapy sessions remained the same. The study concluded that the 2-week program could be considered non-inferior to the 3-week program in both clinical outcomes and satisfaction (Held et al., 2023). The study does not report the prevalence of comorbidity disorders in this veteran sample. It is possible that, in patient groups like ours, with high levels of comorbidity, factors like psychoeducation and physical activity are more important and that a somewhat longer admission with more breaks could be beneficial. Another of our findings is that patients in our sample profited from different aspects of the treatment program. It is thus not self-evident as to which part of the treatment program is superfluous and should be omitted, though Van Woudenberg et al. (2018) in the original PSYTREC program omitted PE listening “due to the intensive treatment format”. It could be argued that an intensive treatment program should be more individualized, so elements that appear non-beneficial to individual patients should be replaced by more beneficial treatment elements; this finding, however, opens up to the potential pitfall of going along with patients' avoidance patterns.

### 4.3 Experienced change

Neither of the two earlier qualitative studies of intensive trauma therapy focused on patient's lived experience of change after completing treatment, though Thoresen et al. (2022) report patients feeling calmer and more present as well as having more hope for the future. In the current study, we found that all participants expressed a change in how they related to their trauma history and that the program had facilitated new insights into their emotional and/or cognitive reaction patterns. All participants reported being more emotionally connected, with less numbness and dissociation. Most further described greater acceptance and ownership of their trauma history and being clearer about not being responsible for traumatic events that they previously had blamed themselves for. In the wake of these insights came not only more self-compassion but also grief as most participants gained a new perspective on their upbringing and past or current relations. Similarly, reductions in maladaptive posttraumatic cognitions (related to among others self-blame) and trauma-affected erroneous perceptions of the self and the world have previously been described as a result of non-intensive trauma-focused treatment (Kangaslampi and Peltonen, 2022; Gjerstad et al., 2024). For some, new perspectives on their upbringing and relations made them change how they wanted to relate to or interact with these relations, some experiencing a need to change relationship patterns or break contact. Viewing their non-professional support system as scanter than anticipated, some of our patients felt lonelier and more vulnerable. This is a potential effect of therapy that we need to be aware of. Patients may need an updated post-discharge plan where they are adequately supported in what our sample describes as a demanding transition.

Thoresen et al. (2022) described the elements of the treatment program (EMDR and PE) as beneficial as they were effective in different ways. In the current study, we found that some patients described a synergistic effect between treatment modalities and that the elements of PE and EMDR complemented each other, making the combined effect stronger. This finding is interesting as the two approaches differ somewhat in their therapeutic target; PE's main mechanism of change appears to be through changing dysfunctional attitudes and thought patterns (cognitive change) (Kangaslampi and Peltonen, 2022; Brown et al., 2019), while the most supported mechanism of change in EMDR is through reducing vividness and emotionality of trauma memories as well as through changed physiological parameters (Landin-Romero et al., 2018; Wadji et al., 2022). As suggested by Van Minnen et al. (2018), it seems possible that treating patients' trauma histories from several angles within the same treatment program could create a fuller, more multifaceted therapeutic trauma processing.

### 4.4 Strengths and limitations

This study has some important limitations. It consists of female participants only, which limits its generalizability. Due to technical issues with the recording, one interview could not be included in the data set and thematic analysis. This unfortunate omission could have potentially somewhat influenced our findings. The smaller number of participants in our study is compensated for by the format of our interview and the fact that the study explores a novel therapy intervention that has received very scarce previous qualitative investigation. The interviews were carried out shortly after the treatment was completed. The participants thus remembered the treatment vividly. Not enough time had passed, however, to explore the long-term effect of the program.

## 5 Conclusion and recommendations

This study aimed to explore the lived experience of participants in an intensive inpatient trauma treatment program. Our findings suggest that establishing therapeutic alliances within the framework of the treatment program as well as intensive multimodal trauma treatment may contribute to experienced change. Participants described greater acceptance and ownership of their trauma history and greater emotional accessibility as well as changed interpersonal relations. The intensity of the program was described as exhausting, but necessary. Most patients reported at times being too overwhelmed to benefit from elements of the program. Our results thus prompt us to question the optimal level of intensity.

Clinical implications: Our findings suggest that it is important to not only prepare participants adequately before commencing treatment but also better prepare for discharge, which could be particularly important in patients with high levels of comorbidities.

Future research recommendations: Future qualitative studies should interview patients after a longer time interval to investigate if the experienced change from ITTP is lasting. Qualitative exploration of the experience of ITTP therapists could also further improve our knowledge in this field. The optimal level of intensity in intensive trauma treatment should be further explored through qualitative as well as quantitative studies.

## Data availability statement

The raw data supporting the conclusions of this article will be made available by the authors, without undue reservation.

## Ethics statement

The studies involving humans were approved by the Regional Committee for Medical and Health Research Ethics, South East Norway, Section A (REC) has approved the study protocol (reference number; REC South East 0704/202). The studies were conducted in accordance with the local legislation and institutional requirements. The participants provided their written informed consent to participate in this study.

## Author contributions

VV-K: Conceptualization, Formal analysis, Project administration, Writing – original draft. JE: Conceptualization, Data curation, Formal analysis, Methodology, Project administration, Writing – review & editing. MJ: Conceptualization, Formal analysis, Writing – review & editing, Supervision. WA: Conceptualization, Project administration, Writing – review & editing. JE: Conceptualization, Formal Analysis, Methodology, Project administration, Writing – original draft.

## References

[B1] Alvesson M. Deetz S. A. Alvesson M. (2020). Doing Critical Research. [Second edition]. ed. Thousand Oaks, CA: SAGE Publications. 10.4135/9781529682649

[B2] Auren T. J. B. Klæth J. R. Jensen A. G. Solem S. (2022). Intensive outpatient treatment for PTSD: an open trial combining prolonged exposure therapy, EMDR, and physical activity. Eur. J. Psychotraumatol. 13:2128048. 10.1080/20008066.2022.2128048 36237826 PMC9553174

[B3] Bisson J. I. Cosgrove S. Lewis C. Roberts N. P. (2015). Post-traumatic stress disorder. BMJ 351:h6161. 10.1136/bmj.h6161 26611143 PMC4663500

[B4] Bisson J. I. Roberts N. P. Andrew M. Cooper R. Lewis C. (2013). Psychological therapies for chronic post-traumatic stress disorder (PTSD) in adults. Cochrane Database Syst. Rev. 2013:Cd003388. 10.1002/14651858.CD003388.pub4 24338345 PMC6991463

[B5] Bradley R. Greene J. Russ E. Dutra L. Westen D. (2005). A multidimensional meta-analysis of psychotherapy for PTSD. Am. J. Psychiatry. 162, 214–227. 10.1176/appi.ajp.162.2.214 15677582

[B6] Braun V. Clarke V. (2006). Using thematic analysis in psychology. Qualit. Res. Psychol. 3, 77–101. 10.1191/1478088706qp063oa32100154

[B7] Brown L. A. Zandberg L. J. Foa E. B. (2019). Mechanisms of change in prolonged exposure therapy for PTSD: implications for clinical practice. J. Psychother. Integr. 29, 6–14. 10.1037/int000010927357964

[B8] Brynhildsvoll Auren T. J. Gjerde Jensen A. Rendum Klæth J. Maksic E. Solem S. (2021). Intensive outpatient treatment for PTSD: a pilot feasibility study combining prolonged exposure therapy, EMDR, physical activity, and psychoeducation. Eur. J. Psychotraumatol. 12:1917878. 10.1080/20008198.2021.1917878 34025928 PMC8128113

[B9] Castleberry A. Nolen A. (2018). Thematic analysis of qualitative research data: is it as easy as it sounds? Curr. Pharm. Teach. Learn. 10, 807–815. 10.1016/j.cptl.2018.03.019 30025784

[B10] Clarke V. Braun V. Hayfield N. (2015). “Qualitative psychology: a practical guide to research methods,” in 3rd ed. J. A. Smith (Los Angeles: Sage).

[B11] Cusack K. Jonas D. E. Forneris C. A. Wines C. Sonis J. Middleton J. C.. (2016). Psychological treatments for adults with posttraumatic stress disorder: a systematic review and meta-analysis. Clin. Psychol. Rev. 43, 128–141. 10.1016/j.cpr.2015.10.003 26574151

[B12] Edmond T. Sloan L. McCarty D. (2004). Sexual abuse survivors' perceptions of the effectiveness of EMDR and eclectic therapy. Res. Soc. Work Pract. 14, 259–272. 10.1177/1049731504265830

[B13] Flückiger C. Del Re A. C. Wlodasch D. Horvath A. O. Solomonov N. Wampold B. E.. (2020). Assessing the alliance-outcome association adjusted for patient characteristics and treatment processes: a meta-analytic summary of direct comparisons. J. Couns Psychol. 67, 706–711. 10.1037/cou0000424 32212755 PMC7529648

[B14] Foa E. B. Hembree E. A. Rauch S. A. M. Rothbaum B. O. Haukeland E. (2021). Prolonged Exposure Therapy for PTSD: Emosjonell Prosessering Av Traumatiske Erfaringer. 1. utgave. ed. Oslo: Gyldendal.

[B15] Gadamer H.- G. (2013). Truth and Method. 1st ed. ed. London, UK: Bloomsbury Academic.

[B16] Gahnfelt H. Carlsson P. F. G. Blomdahl C. (2023). Is it safe enough? A pilot feasibility study of an 8-day intensive treatment for severe PTSD. Front. Psychiat. 14:1200411. 10.3389/fpsyt.2023.1200411 37547221 PMC10397389

[B17] Galovski T. E. Werner K. B. Weaver T. L. Morris K. L. Dondanville K. A. Nanney J.. (2022). Massed cognitive processing therapy for posttraumatic stress disorder in women survivors of intimate partner violence. Psychol. Trauma. 14, 769–779. 10.1037/tra0001100 34472941

[B18] Gjerstad S. F. Nordin L. Poulsen S. Spadaro E. F. A. Palic S. (2024). How is trauma-focused therapy experienced by adults with PTSD? A systematic review of qualitative studies. BMC Psychol. 12:135. 10.1186/s40359-024-01588-x 38459602 PMC10924413

[B19] Goldfried M. R. (1980). Toward the delineation of therapeutic change principles. Am. Psychol. 35, 991–999. 10.1037/0003-066X.35.11.991 7436119

[B20] Hase M. Brisch K. H. (2022). The therapeutic relationship in EMDR therapy. Front. Psychol. 13:835470. 10.3389/fpsyg.2022.835470 35712194 PMC9197431

[B21] Held P. Smith D. L. Pridgen S. Coleman J. A. Klassen B. J. (2023). More is not always better: 2 weeks of intensive cognitive processing therapy-based treatment are noninferior to 3 weeks. Psychol. Trauma. 15, 100–109. 10.1037/tra0001257 36656744 PMC10258911

[B22] Hill C. E. Knox S. Thompson B. J. Williams E. N. Hess S. A. Ladany N.. (2005). Consensual qualitative research: an update. J. Counsel. Psychol. 52, 196–205. 10.1037/0022-0167.52.2.196

[B23] Howard R. Berry K. Haddock G. (2022). Therapeutic alliance in psychological therapy for posttraumatic stress disorder: a systematic review and meta-analysis. Clin. Psychol. Psychother. 29, 373–399. 10.1002/cpp.2642 34237173

[B24] Kangaslampi S. Peltonen K. (2022). Mechanisms of change in psychological interventions for posttraumatic stress symptoms: a systematic review with recommendations. Curr. Psychol. 41, 258–75. 10.1007/s12144-019-00478-527532144

[B25] Kessler R. C. Aguilar-Gaxiola S. Alonso J. Benjet C. Bromet E. J. Cardoso G.. (2017). Trauma and PTSD in the WHO world mental health surveys. Eur. J. Psychotraumatol. 8:1353383. 10.1080/20008198.2017.1353383 29075426 PMC5632781

[B26] Kolthof K. A. Voorendonk E. M. Van Minnen A. De Jongh A. (2022). Effects of intensive trauma-focused treatment of individuals with both post-traumatic stress disorder and borderline personality disorder. Eur. J. Psychotraumatol. 13:2143076. 10.1080/20008066.2022.2143076 PMC9704092 38872595

[B27] Kovacevic M. Tharaud J. B. Montes M. Mundle R. S. Splaine C. C. Silverberg J.. (2023). 'Undoing a knot': a qualitative study of massed 1-week cognitive processing therapy. Eur. J. Psychotraumatol. 14:2205126. 10.1080/20008066.2023.2205126 37288955 PMC10251796

[B28] Kvale S. Brinkmann S. (2009). InterViews: Learning the Craft of Qualitative Research Interviewing, 2nd ed. Thousand Oaks, CA, US: Sage Publications, Inc. xviii, 354-xviii.

[B29] Landin-Romero R. Moreno-Alcazar A. Pagani M. Amann B. L. (2018). How does eye movement desensitization and reprocessing therapy work? A systematic review on suggested mechanisms of action. Front Psychol. 9:1395. 10.3389/fpsyg.2018.01395 30166975 PMC6106867

[B30] Lewis C. Roberts N. P. Andrew M. Starling E. Bisson J. I. (2020a). Psychological therapies for post-traumatic stress disorder in adults: systematic review and meta-analysis. Eur. J. Psychotraumatol. 11:1729633. 10.1080/20008198.2020.1729633 32284821 PMC7144187

[B31] Lewis C. Roberts N. P. Gibson S. Bisson J. I. (2020b). Dropout from psychological therapies for post-traumatic stress disorder (PTSD) in adults: systematic review and meta-analysis. Eur. J. Psychotraumatol. 11:1709709. 10.1080/20008198.2019.1709709 32284816 PMC7144189

[B32] Malterud K. (2011). Kvalitative Metoder i Medisinsk Forskning: En Innføring. 3. utg. ed. Oslo: Universitetsforl.

[B33] Marich J. Dekker D. Riley M. O'Brien A. (2020). Qualitative research in EMDR therapy: exploring the individual experience of the how and why. J. EMDR Pract. Res. 14:EMDR-D. 10.1891/EMDR-D-20-0000111261958

[B34] Paridaen P. Voorendonk E. M. Gomon G. Hoogendoorn E. A. van Minnen A. de Jongh A.. (2023). Changes in comorbid depression following intensive trauma-focused treatment for PTSD and complex PTSD. Eur. J. Psychotraumatol. 14:2258313. 10.1080/20008066.2023.2258313 37796651 PMC10557564

[B35] Patton M. Q. (1990). Qualitative Evaluation and Research Methods, 2nd ed. Thousand Oaks, CA, US: Sage Publications, Inc., 532.

[B36] Qu S. Dumay J. (2011). The qualitative research interview. Qualit. Res. Account. Manag. 8, 238–264. 10.1108/11766091111162070

[B37] Sciarrino N. A. Warnecke A. J. Teng E. J. (2020). A systematic review of intensive empirically supported treatments for posttraumatic stress disorder. J. Trauma Stress. 33, 443–454. 10.1002/jts.22556 32598561

[B38] Shapiro F. (2018). Eye Movement Desensitization and Reprocessing (EDMR) Therapy: Basic Principles, Protocols, and Procedures. Third edition. ed. New York: The Guilford Press.

[B39] Sheehan D. V. Lecrubier Y. Sheehan K. H. Amorim P. Janavs J. Weiller E.. (1998). The Mini-International Neuropsychiatric Interview (M.I.N.I.): the development and validation of a structured diagnostic psychiatric interview for DSM-IV and ICD-10. J. Clin. Psychiat. 59, 22–33. 10.1037/t18597-000 9881538

[B40] Sherrill A. M. Maples-Keller J. L. Yasinski C. W. Loucks L. A. Rothbaum B. O. Rauch S. A. M.. (2022). Perceived benefits and drawbacks of massed prolonged exposure: a qualitative thematic analysis of reactions from treatment completers. Psychol. Trauma. 14, 862–870. 10.1037/tra0000548 31971423

[B41] Thoresen I. H. Auren T. J. B. Langvik E. O. Engesæth C. Jensen A. G. Klæth J. R.. (2022). Intensive outpatient treatment for post-traumatic stress disorder: a thematic analysis of patient experience. Eur. J. Psychotraumatol. 13:2043639. 10.1080/20008198.2022.2043639 35479299 PMC9037168

[B42] Tong A. Sainsbury P. Craig J. (2007). Consolidated criteria for reporting qualitative research (COREQ): a 32-item checklist for interviews and focus groups. Int. J. Qual Health Care. 19, 349–357. 10.1093/intqhc/mzm042 17872937

[B43] Van Minnen A. Hendriks L. Kleine R. Hendriks G. J. Verhagen M. De Jongh A.. (2018). Therapist rotation: a novel approach for implementation of trauma-focused treatment in post-traumatic stress disorder. Eur. J. Psychotraumatol. 9:1492836. 10.1080/20008198.2018.1492836 30034642 PMC6052418

[B44] Van Woudenberg C. Voorendonk E. M. Bongaerts H. Zoet H. A. Verhagen M. Lee C. W.. (2018). Effectiveness of an intensive treatment programme combining prolonged exposure and eye movement desensitization and reprocessing for severe post-traumatic stress disorder. Eur. J. Psychotraumatol. 9:1487225. 10.1080/20008198.2018.1487225 30013726 PMC6041781

[B45] Wadji D. L. Martin-Soelch C. Camos V. (2022). Can working memory account for EMDR efficacy in PTSD? BMC Psychol. 10:245. 10.1186/s40359-022-00951-0 36320044 PMC9623920

[B46] Watson P. (2019). PTSD as a public mental health priority. Curr. Psychiat. Rep. 21:61. 10.1007/s11920-019-1032-1 31243637

[B47] Whitehouse J. (2021). What do clients say about their experiences of EMDR in the research literature? A systematic review and thematic synthesis of qualitative research papers. Eur. J. Trauma Dissoc. 5:100104. 10.1016/j.ejtd.2019.03.002

[B48] WHO (2013). Guidelines for the management of conditions specifically related to stress. Geneva: World Health Organization. Available online at: https://www.who.int/news/item/06-08-2013-who-releases-guidance-on-mental-health-care-after-trauma#New%20Care%20Protocols%20For%20Post-Traumatic%20Stress%20Disorder%20and%20Others (accessed October 15, 2023). 24049868

[B49] Williams J. B. Gibbon M. First M. B. Spitzer R. L. Davies M. Borus J.. (1992). The structured clinical interview for DSM-III-R (SCID). II. Multisite test-retest reliability. Arch. Gen. Psychiatry. 49, 630–636. 10.1001/archpsyc.1992.01820080038006 1637253

[B50] Zoet H. A. Wagenmans A. van Minnen A. de Jongh A. (2018). Presence of the dissociative subtype of PTSD does not moderate the outcome of intensive trauma-focused treatment for PTSD. Eur. J. Psychotraumatol. 9:1468707. 10.1080/20008198.2018.1468707 29805779 PMC5965028

